# mCSM-PPI2: predicting the effects of mutations on protein–protein interactions

**DOI:** 10.1093/nar/gkz383

**Published:** 2019-05-22

**Authors:** Carlos H M Rodrigues, Yoochan Myung, Douglas E V Pires, David B Ascher

**Affiliations:** 1 Department of Biochemistry and Molecular Biology, University of Melbourne, Melbourne, Australia; 2 ACRF Facility for Innovative Cancer Drug Discovery, Bio21 Institute, University of Melbourne, Melbourne, Australia; 3 Structural Biology and Bioinformatics, Baker Heart and Diabetes Institute, Melbourne, Australia; 4 Department of Biochemistry, University of Cambridge, Cambridge, UK

## Abstract

Protein–protein Interactions are involved in most fundamental biological processes, with disease causing mutations enriched at their interfaces. Here we present mCSM-PPI2, a novel machine learning computational tool designed to more accurately predict the effects of missense mutations on protein–protein interaction binding affinity. mCSM-PPI2 uses graph-based structural signatures to model effects of variations on the inter-residue interaction network, evolutionary information, complex network metrics and energetic terms to generate an optimised predictor. We demonstrate that our method outperforms previous methods, ranking first among 26 others on CAPRI blind tests. mCSM-PPI2 is freely available as a user friendly webserver at http://biosig.unimelb.edu.au/mcsm_ppi2/.

## INTRODUCTION

Most biological processes, including cell proliferation (1), signalling (2), host-pathogen interactions (3) and protein transport (4), are intrinsically coordinated through complex networks of protein–protein interactions. The diversity and size of the interactome offers a highly selective and tunable way to modulate protein activities and pathways (5). Genetic variations leading to changes in the binding affinity of these interactions can disrupt or directly affect the formation of interacting complexes and consequently lead to disease (6–16) and drug resistance (17–19).

Advances in next-generation sequencing techniques have created an explosive increase in the number of genetic variants available in the literature. However, experimental techniques to study these variants are still expensive and time consuming. mCSM (20) was one of the first scalable computational tools to accurately predict the effects of mutations on binding affinity. Previous methods were limited either in terms of their throughput (21,22) or in terms of their performance (23). Since then, significant efforts have been devoted to computationally study the effects of mutations on protein complexes (24,25) but their poor predictive performance on new variants, particularly mutations that lead to increased binding affinity of the complex, has limited their use. In addition, the increase in amount of experimental evidence of effects of variants on binding affinity offers the opportunity to develop new and more accurate methods.

Our previously described graph-based signatures concept has proven to be a powerful approach and has been widely applied to the study of protein structure, including how mutations alter protein stability (20,26), dynamics (27) and interactions with other molecules (20,28–34).

Here we introduce mCSM-PPI2, a webserver that integrates our well-established mCSM graph-based based signatures framework with evolutionary information, inter-residue non-covalent interaction networks analysis and energetic terms, in order to provide an optimized overall prediction performance.

## MATERIALS AND METHODS

### Data sets

The data used on this work was derived from the recently updated version of the SKEMPI database (35), which compiles experimental data on changes in thermodynamic and kinetic parameters on mutation for protein–protein complexes that have 3D structures deposited in the PDB. SKEMPI 2.0 (36) includes new mutations identified in the literature after its first release, including data available from three other databases: ABbind (37), PROXiMATE (38) and dbMPIKT (39). The average mutation effect was considered for variants reported in multiple experiments when these varied by less than 2.0 kcal/mol and discarded otherwise. After filtering for only single-point mutations with available experimental crystal structures of the wild-type, we were able to collect 4169 (S4169) variants in 319 different complexes. All protein structures were collected from the Protein Data Bank and a series of pre-processing steps were performed to account for the diversity of structures (see Supplementary material).

The binding affinity of protein–protein complexes were used to calculate the binding Gibbs free energy (Δ*G*):

\begin{equation*}\Delta G\ = \ RT\ln\left( {{K_{\rm D}}} \right)\end{equation*}

where $R\ = \ 1.985\times \ {10^{ - 3}}\ {\rm kcal}\ {{\rm K}^{ - 1}}{{\rm mol}^{ - 1}}$ is the ideal gas constant, T is the temperature (in K) and ${K_{\rm D}}$ is the equilibrium dissociation constant of the protein–protein complex (in molar). The change in binding affinity upon mutation was calculated as follows:

\begin{equation*}\Delta \Delta {G_{{\rm wt - mt}}}\ = \ \Delta {G_{{\rm wild - type}}} - \ \Delta {G_{{\rm mutant}}}\end{equation*}

Since the Gibbs free energy formulation is a thermodynamic state function a change in binding affinity of a mutation from a wild-type protein to its mutant (ΔΔ*G*_WT→MT_) should be equivalent to the negative change binding free energy of the hypothetical reverse mutation, from the mutant to the wild-type protein (–ΔΔ*G*_MT→WT_) (40). Given the unbalanced nature of the original dataset collected from SKEMPI 2.0, 901 variants increased (ΔΔ*G*_wt-mt_ ≥ 0) and 3268 decreased (ΔΔ*G*_wt-mt_ < 0) binding affinity, and in order to build a more robust and balanced predictive method, we also included the hypothetical reverse mutations. Therefore, the final dataset for building mCSM-PPI2 predictive model includes 8338 single-point mutations (S8338), which represents an increase of up to three-fold in datapoints in comparison with previous methods that used data from the first version of SKEMPI with 2007 (S2007) (20,23), 1964 (S1964) (25), 1102 (S1102) (24) and 1327 (S1327) mutations (41).

A subset of 487 mutations in 56 complexes (S487) contained within S4169 and not in S2007 were recently curated (24) and here we used as evidence to evaluate the performance of mCSM-PPI2. A summary of different subsets derived from SKEMPI is shown in Supplementary Table S1.

The datasets used in this work are freely available for download at http://biosig.unimelb.edu.au/mcsm_ppi2/datasets.

### Graph-based structural signatures

mCSM-PPI2 uses as one of its core components our well established graph-based structural signatures (mCSM) to represent the environment of the wild-type residue. This approach models both the geometry and physicochemical properties of the interactions and architecture of wild-type structure and has been widely applied to the study of small molecule and protein structure (20,26–34,42). Our signatures represent atoms as nodes and their interactions as edges, with their physicochemical properties encoded based upon the amino acid residue properties, denoted by a pharmacophore. From this representation of the residue environment, distance patterns between atoms characterized by their properties are summarized in concise signatures as cumulative distribution functions.

### Modelling effects of mutation

**Figure 1.**
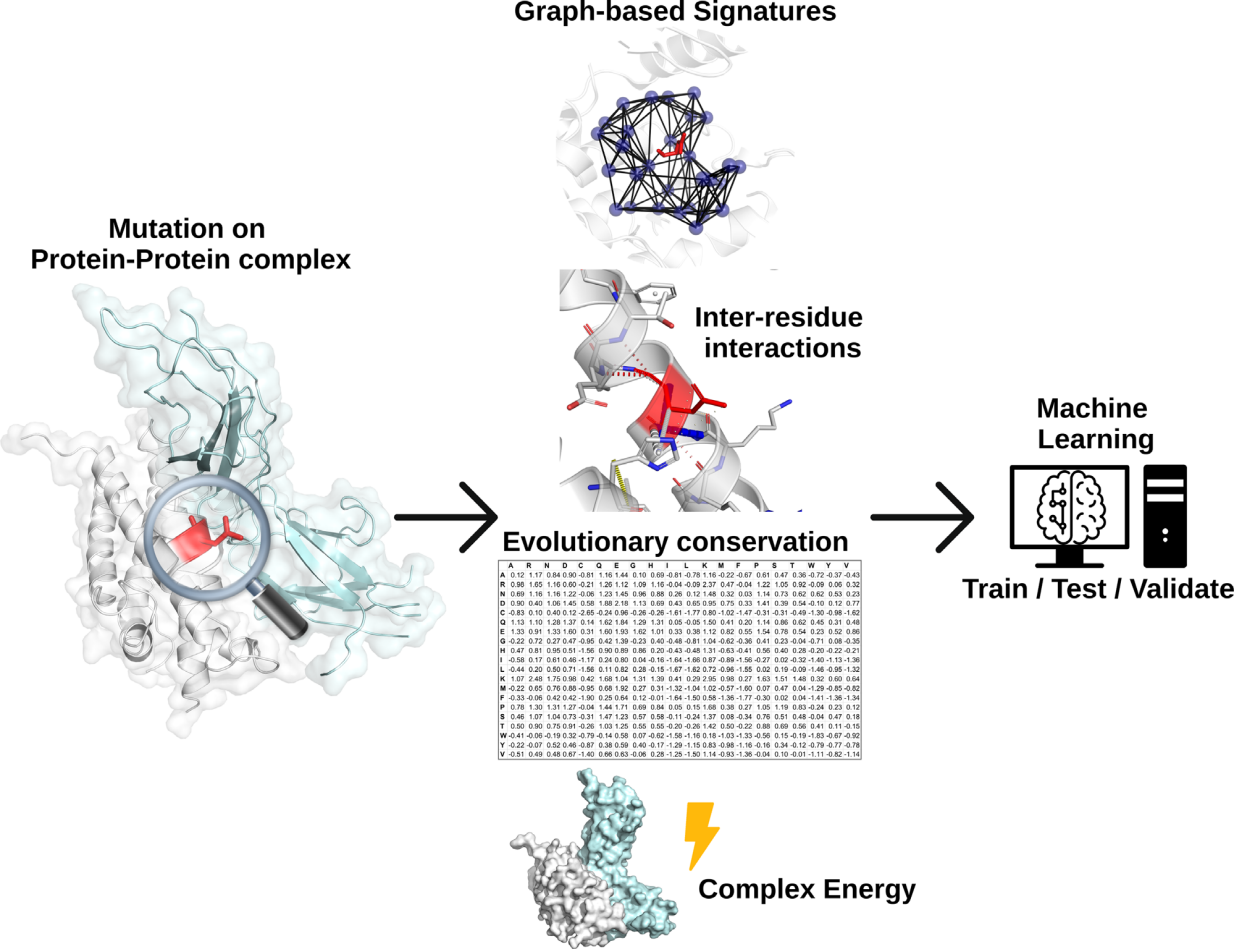
mCSM-PPI2 methodology workflow. The method relies on graph-based signatures, which model distance patterns and encode geometrical and physico-chemical properties on wild-type residue environment. Network analysis based on non-covalent interactions of wild-type residue and interacting interface along with evolutionary information and energy terms are also used. All features are used as evidence to train, test and validate machine learning algorithms.

Single-point mutations can affect protein–protein interactions via different molecular mechanisms, including changing folding free energy of interacting partners or disrupting non-covalent interactions essential for complex formation (6,43). In mCSM-PPI2, we have included six new distinct types of features that were not used in our first method (Supplementary Table S2). These were combined with our well-established graph-based signatures as evidence for training a machine learning algorithm (see Supplementary material) to better explore the effects of mutations in protein–protein binding affinity (Figure 1).

#### Wild-type residue environment

Based on 3D structures collected from the Protein Data Bank (44), we were able to calculate Relative Solvent Accessibility (RSA), torsion angle PHI and residue depth for the wild-type residue using BioPython (45) version 1.7. We also extracted information on the amino acid content in the sequence of the chain in which the mutation occurs using iFeature (46).

#### Nature of wild-type and mutant residues

The conformational flexibility of glycine side chains and the rigidity of proline side chains are important for defining the backbone flexibility. Mutations from and to these two amino acids can lead to large structural effects. For our model we included binary terms to capture if the mutation was from or to a glycine or proline.

#### Evolutionary information

Binding regions are known to be evolutionarily conserved, which has been exploited in a variety of studies to identify potential protein interaction interfaces. For mCSM-PPI2 we also harnessed this information by using the Position Specific Scoring Matrix (PSSM) scores. PSSM was calculated through PSI-BLAST of BLAST 2.2.3 using the non-redundant Swiss-Prot database of protein sequences and the sequence of the chain in which the mutation occurs as the query parameter.

#### Non-covalent interaction network analysis

We performed analysis of the non-covalent interactions for the wild-type residue and for the closest interface using the contacts calculated by Arpeggio (47). Here, we extracted two types of information: the difference between the number of contacts of wild-type and mutant residue for covalent, Van der Waals’, aromatic and hydrogen bond contacts, and complex network metrics for the contact graph of the closest interface of interaction, from which we extracted centrality metrics, including closeness and central points (48). In this work, we consider a residue to be at an interaction interface if it is located at most 5 Å away from the interacting partner, following previous studies. In addition, we included three protein contact potentials scores from the AAindex database (49) (Supplementary Table S3).

#### Energetic terms

Interaction energy information between the two interacting chains were extracted from FoldX (22). In addition, we included the predicted folding free energy change upon mutation.

#### Atomic fluctuation

We used the Bio3D R package (50) to calculate the atomic fluctuations of the structure of the monomer where the mutation occur using calpha and pfanm force fields to account for effects on protein flexibility/rigidity.

## WEBSERVER

We have implemented mCSM-PPI2 as a user-friendly and freely available webserver (http://biosig.unimelb.edu.au/mcsm_ppi2/). The server front end was built using Materialize framework version 1.0.0, while the back-end was built in Python via the Flask framework (Version 1.0.2). It is hosted on a Linux server running Apache.

### Input

mCSM-PPI2 can be used in two different ways: to either assess the effects of mutations specified by the user input or to predict the effects of mutations at the protein–protein interface in an automated manner. For user-specified variations two options are available (Supplementary Figure S1). The ‘Single Mutation’ option requires one to provide a PDB file or PDB accession code of the structure of the protein complex, the point mutation specified as a string containing the wild-type residue one-letter code, its corresponding residue number and the mutant residue one-letter code. The ‘Mutation List’ option allows users to upload a list of mutations in a plain text file for batch processing. For both options, users are also required to specify the chain identifier in which the wild-type residues are located.

Alternatively, for assessing effects of mutations at protein–protein interfaces the server requires the user to provide a PDB file or PDB accession code and select one of two options: alanine scanning (all interface residues are mutated to an Alanine) or saturation mutagenesis (all interface residues are mutated to every other amino acid) (Supplementary Figure S2).

In order to assist users to submit their jobs for predictions, sample submission entries are available in both submission pages and a help page is also available via the top navigation bar.

### Output

For the Single Mutation option (Supplementary Figure S3), mCSM-PPI2 outputs the predicted change in binding affinity (in kcal/mol) along with an interactive 3D viewer, built using NGL viewer (51), showing non-covalent interactions, generated with Arpeggio, at the mutated position. A set of controllers are available for users to hide and show the different types of interactions and to alternate between wild-type and mutant structures. In addition, a 2D viewer displaying non-covalent interactions of wild-type and mutant structures is also shown. Pymol sessions are available for download. For the Mutation List option (Supplementary Figure S4), the results are summarized in a downloadable table from which users can access details for each single variant.

For the Alanine Scanning option on the interface analysis, the server first presents a table with all the interfaces identified on the submitted structure, and it also allows for inspection of the individual interfaces. On the results page of each interface the server shows a downloadable table with the prediction outcomes for each mutation, a bar chart that summarizes the predicted changes in binding affinity (Supplementary Figure S5) and an interactive 3D viewer in which the residues are coloured according to the predicted value (Supplementary Figure S6). Similarly, for the Saturation Mutagenesis option, mCSM-PPI2 outputs a table with all the interfaces identified and allows the users to access detailed information on each interface. For each interface, the server outputs a table compiling the results for all variants (Supplementary Figure S7), a heatmap of all interface residues and their respective mutations (Supplementary Figure S8), and a 3D viewer in which the residues are coloured according to the average prediction for each particular position (Supplementary Figure S9).

## VALIDATION

### Performance on cross-validation

**Figure 2.**
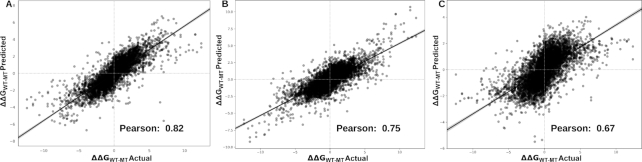
Performance evaluation on cross-validation. mCSM-PPI2 was able to achieve a Pearson's correlation of 0.82 and RMSE 1.18 kcal/mol when trained on the S8338 dataset applying 10-fold cross-validation 10 times (**A**). In low-redundancy sets, mCSM-PPI2 was able to achieve a correlation of 0.75 and 0.67 on leave-one-complex-out (**B**) and leave-one-binding-site-out (**C**), respectively.

In order to build a more robust and reliable predictive model we performed four types of validation. Firstly, we performed 10-times stratified 10-fold cross-validation, using 90% of our original dataset (S8338) for training and the remaining as a blind test. Selection of the blind test was repeated 10 times in a stratified manner, with the model retrained on the remaining data, in order to test the robustness of the model (see Supplementary material). For this approach the hypothetical reverse mutations were kept in either training or test sets during the splits according to its counterpart original mutation. Our method was able to achieve an average Pearson correlation (ρ) of 0.82 with a standard deviation (σ) of 0.06 across the 10 runs (Figure 2A) showing a more balanced prediction when distinguishing between mutations that increase binding affinity from decreasing ones than other methods (Supplementary Table S4). We also evaluate the performance of mCSM-PPI2 when trained only on the original subset of mutations from SKEMPI2 (S4169) using the same procedure and obtained a correlation of 0.76 and RMSE of 1.19 kcal/mol. These results corroborate the use of reverse mutations in order to improve performance and robustness of our predictive model. Performance comparison between mCSM-PPI2 and other methods on different versions of SKEMPI and performance of individual types of attributes are shown in Supplementary Tables S5 and S6, respectively.

We further evaluated the performance of our approach on two low-redundancy sets; low redundant at the (i) complex and (ii) interface level. The complex low redundancy test was performed using leave-one-complex out cross-validation, in which we trained our model on 318 complexes of our dataset and evaluate the performance on the one remaining complex. After repeating this procedure for each complex we achieved ρ = 0.75 (Figure 2B) and Root Mean Square Error (RMSE) of 1.30 kcal/mol, outperforming MutaBind (25) (ρ = 0.68 and RMSE = 1.57 kcal/mol).

Similarly, we applied leave-one binding site out using the ‘hold-out’ information extracted from SKEMPI2. Here, we removed all mutations located in identical binding sites for testing and trained on the remaining data. mCSM-PPI2 achieved ρ = 0.67 (RMSE = 1.39 kcal/mol) (Figure 2C), which was significantly higher (p-value < 0.0001 by Fisher *r*-to-*z* transformation) than the results reported for MutaBind when trained using only mutations from SKEMPI1 (ρ = 0.57 and RMSE = 1.57 kcal/mol).

In addition, we evaluated the performance of our approach on a subset of 472 mutations (S472) not present within the first version of SKEMPI but included in SKEMPI2. For this experiment, we trained a predictive model using all variants from the first version of SKEMPI (S1964). Our method achieved a correlation of 0.63 (RMSE = 1.11 kcal/mol).

### Validation on CAPRI

mCSM-PPI2 was further validated against the CAPRI (52) round 26, which is composed of 1862 experimentally characterised mutations in two *de novo* influenza inhibitor targets (T55 and T56: 1007 mutations at 53 different positions in T55 and 855 mutations at 45 different positions in T56). The *in vitro* experimental measurements used the enrichment values generated from deep sequencing and were calculated based on the binary logarithm of the ratio of number of times the variant sequence was observed after and before the selection for binding. Although the 3D structures for these two complexes were not available, structures of close homologues have been described (53,54) and were used for generating homology model structures by introducing point mutations using Modeller (55) (see Supplementary Materials). mCSM-PPI2 was able to achieve a Kendall's score of up to 0.42 and 0.32 for mutations in T55 and T56, respectively, ranking first amongst 26 other methods (Supplementary Figure S10 and Table 1).

**Table 1.** Comparative performance of mCSM-PPI2 on CAPRI and the blind test set for the complex MDM2-P53

	CAPRI (T55)		CAPRI (T56)		MDM2-P53	
Method	Kendall	RMSE (kcal/mol)	Kendall	RMSE (kcal/mol)	*ρ*	RMSE (kcal/mol)
**mCSM-PPI2**	**0.42**	**2.55**	**0.32**	**4.06**	**0.40**	**0.36**
mCSM (20)	0.16**	3.71	0.13**	4.15	0.23	0.83
MutaBind (25)	0.41	2.58	0.30	4.27	*NA*	*NA*
iSEE (24)	*NA*	*NA*	*NA*	*NA*	0.24	0.81
BeAtMuSiC (23)	0.28**	3.04	0.30	4.06	−0.23*	0.91
FoldX (22)	0.12**	3.94	0.16**	4.33	−0.14*	
		0.90				
MMPBSA (21)	0.19**	5.40	0.08**	28.04	*NA*	*NA*

*p-value < 0.05 by Fisher r-to-z transformation test compared to mCSM-PPI2

**p-value < 0.05 by transforming tau-to-r followed by Fisher r-to-z transformation. *NA*: Data not available.

### Blind test

The performance of mCSM-PPI2 was further evaluated on a small set of 26 variants at the interface of interaction of the MDM2-p53 complex (PDB 1YCR) (24). Our method achieved a Pearson's Correlation of 0.40 and an RMSE = 0.36 kcal/mol outperforming mCSM, iSEE, FoldX, BeAtMuSiC (23) (Table 1).

Finally, we looked at the ability of mCSM-PPI2 to accurately identify PPI hotspots, residues that contribute to the majority of the binding free energy of the interaction and have been recognized as important sites for drug development (5). Here we evaluated the performance of mCSM-PPI2 across a previously proposed set of 378 alanine-scanning experimental mutations within 19 different protein–protein complexes (56,57) (Supplementary Table S7). In order to minimize biases, for this experiment we removed 232 variants from S8338 which were redundant with our set of 378 alanine scanning mutations. Our predictive model was was able to accurately distinguish hot and not-hot spots (95% of hotspots and 92% of non-hotspots were correctly predicted) outperforming the results reported for Robetta (precision of 79% and 68% when predicting hotspots and non-hotspots, respectively). The predicted changes in binding energy showed that mCSM-PPI2 predictions correlated strongly with the experimental data (Pearson's Correlation of 0.95 and RMSE of 0.25 kcal/mol; Supplementary Figure S11). These results indicate that mCSM-PPI2 could also be a powerful tool for hotspot identification.

## CONCLUSION

Here, we introduce mCSM-PPI2, a web server that implements an integrated computation approach for predicting effects of missense mutations in protein–protein affinity. By consolidating our graph-based signatures framework with evolutionary information, inter-atomic contacts and energy terms our updated method has shown to perform better than its previous version and other methods. In addition, the use of hypothetical reverse mutations has shown to improve the robustness of our predictive model allowing for a more balanced prediction. mCSM-PPI2 is freely available as a user-friendly and easy to use web server at http://biosig.unimelb.edu.au/mcsm_ppi2/.

## Supplementary Material

gkz383_Supplemental_Files
