# Supplementary material for: mCSM-PPI2: predicting the effects of mutations on protein–protein interactions
Source: Nucleic Acids Res. 2019 May 22;47(W1):W338–44. doi: 10.1093/nar/gkz383 (PMC6602427; doi:10.1093/nar/gkz383)
Supplement: gkz383_Supplemental_Files [file gkz383_supplemental_files.docx]

# SUPPLEMENTARY MATERIAL

**mCSM-PPI2: predicting the effects of mutations on protein-protein interactions**

Carlos H.M. Rodrigues^1,2,3^, Yoochan Myung^1,2,3^, Douglas E.V. Pires^1,2,3,^*, David B. Ascher^1,2,3,4,^*

^1^Department of Biochemistry and Molecular Biology, University of Melbourne;

^2^ACRF Facility for Innovative Cancer Drug Discovery, Bio21 Institute, University of Melbourne;

^3^Structural Biology and Bioinformatics, Baker Heart and Diabetes Institute

^4^Department of Biochemistry, University of Cambridge;

*To whom correspondence should be addressed D.B.A. Tel: +61 90354794; Email: david.ascher@unimelb.edu.au or da382@cam.ac.uk. Correspondence may also be addressed to D.E.V.P. douglas.pires@unimelb.edu.au.

# Machine Learning

mCSM-PPI2 was built using the open source module Scikit-Learn version 0.20.1 (1). Scikit-Learn provides a collection of simple and powerful functions, tools and algorithms to perform machine learning and data analysis in Python. Our final model and the results presented in this work were generated based on the ExtraTrees algorithm, a tree-based ensemble method for supervised classification and regression tasks (2). This algorithm operates in a similar manner as the Random Forest algorithm with the difference that decisions boundaries used when splitting the nodes for building the trees are chosen randomly. When building our predictive models we used the default parameters defined in Scikit-Learn except for the number of instances, which was set to 300.

# Stratified Cross-Validation

Results for 10-fold cross-validation performance of mCSM-PPI2 were generated after 10 times repetition of the traditional cross-validation in a stratified manner (pseudo-code below). The dataset used for training was divided in 10 subsets and for each repetition one subset was used for testing and the remaining data was used for training in which 10-fold cross-validation was carried out. Therefore, every subset was used as test set only one time. This procedure was used in order to reduce redundancy over the repetitions.

Pseudo-code for stratified cross-validation:

*N = 10*

*K = 10*

*subsets = split(original_dataset, N)*

*for each subset:*

*remaining_dataset = original_dataset - subset*

*performance_on_crossvalidation(original_dataset - subset, K)*

*model = train_predictive_model(remaining_dataset)*

*model.evaluate(subset)*

# Homology Model Structures for CAPRI Dataset

3D structures for the *de novo* designed influenza inhibitors (HB36.4 and HB80.3) in complex with hemagglutinin (HA), specified on the 26th round of CAPRI as targets T55 and T56, were obtained via homology modelling using Modeller version 9.21 (3) and close homologues previously defined (4,5). The 3D structure for Target 55 were obtained by introducing a single point mutation (N64K) on the structure of HB36.3-HA (PDB: 3R2X). For Target 56, we used the crystal structure of the complex HB80.4-HA (PDB: 4EEF) as the template and introduced 5 point mutations (K12G, I17L, I21L, K35A, K42S).

**Pre-Processing of PDB Structures**

Due to limitations and eccentricities of the PDB format, we performed a series of steps to ensure that structures could be processed by external software. These are described as follows:

- If an accession code for a protein structure on the Protein Data Bank is provided on the input page, mCSM-PPI2 will try to download the first author assigned biological assembly defined in the headers of the official structure. If no code is found (*e.g.*, on NMR structures), mCSM-PPI2 will use the official structure file available on the Protein Data Bank.
- Where multiple models were present in a structure (*e.g.*, on NMR structures), only the first one was maintained, ensuring only a static state was compared when conducting large scale analysis.
- Water molecules, ligands and non-standard compounds were removed.
- For residues presenting multiple occupancy, only the most prevalent one was selected.
- Given that some of the tools used by mCSM-PPI2 have difficulties dealing with structures with insertion codes, temporary files with residues renumbered sequentially (starting from 0) before running our calculations.
- Missing atoms and residues were not modelled.

**TABLES**

**Table S1** – Summary of datasets of experimental information on point mutations and their effects on binding affinity.

| **Dataset** | **Description** | **Reference** |
| --- | --- | --- |
| S4169 | Variants extracted from Skempi2 | (16) |
| S8338 | S4169 plus all reverse mutations | (16) |
| S2007 | 2007 variants extracted from Skempi1 | (10,17,18) |
| S1964 | 1964 variants extracted from Skempi1 | (17,19) |
| S1327 | 1327 variants extracted from Skempi1 | (17,20) |
| S1102 | 1102 variants extracted from Skempi1 | (17,21) |
| S472 | 472 variants contained in S4169 and not in S2007 | (16,21) |
| S378 | Alanine scanning variants | (22) |

**Table S2** – Comparison of features used in mCSM-PPI1 and mCSM-PPI2. The first version of mCSM-PPI used our graph-based signatures to model the environment of the wild-type residue and pharmacophore to account for the effects of physicochemical changes caused by a point mutation as evidence to train a predictive model. In addition to those two types of features, mCSM-PPI2 also includes six new different classes of features that model different effects of single-point variants.

| Type of Feature | Features | Tool | mCSM-PPI1 | mCSM-PPI2 |
| --- | --- | --- | --- | --- |
| Graph-based Signatures | Distance patterns | mCSM (10) | Yes | Yes |
| Pharmacophore Changes | Hydrophobic, Positive, Negative, Hydrogen acceptor, hydrogen donor, aromatic, sulphur and neutral | mCSM (10) | Yes | Yes |
| Wild-type residue Environment | Relative Solvent Accessibility, torsion angle Phi, Residue depth, amino-acid content of chain in which the wild-type resides in percentage: aliphatic, aromatic, positively charged, negatively charged and uncharged | BioPython (11) iFeature (12) | No | Yes |
| Nature of Wild-type and Mutant Residues | Is glycine? Is glycine and has a positive Phi torsion angle? Is proline? | BioPython (11) | No | Yes |
| Evolutionary Information | PSSM Score | Blast 2.2.6 | No | Yes |
| Non-Covalent interaction network metrics | Difference between contacts: Van der Waals’, aromatic and hydrogen bonds. Complex network metrics for the contact graph of the closest interface of interactions: betweenness, authority score, central points, number of edges. Protein contact potentials scores: SIMK990103, MIYS960101 and ZHAC000104 | Arpeggio (13), iGraph, AAindex Database (6) | No | Yes |
| Energetic Terms | Electrostatic interaction between molecules, cost of having a cis peptide bond and Gibbs free energy change | FoldX (14) | No | Yes |
| Atomic Fluctuation | Score using calpha and pfanm force-fields | Bio3D (15) | No | Yes |

**Table S3** - Protein contact potentials scores from the AAindex database (6) used for mCSM-PPI2 as part of its workflow.

| **AAindex Code** | **Description** | **Reference** |
| --- | --- | --- |
| MIYS960101 | Quasichemical energy of transfer of amino acids from water to the protein environment. | (7) |
| SIMK990103 | Distance-dependent statistical potential (contacts within 7.5-10 Å) | (8) |
| ZHAC000104 | Environment-dependent residue contact energies | (9) |

**Table S4** - mCSM-PPI2 performance comparison on classification using two thresholds (0 and 0.2 kcal/mol) in which ΔΔG >= threshold was considered to increase affinity and ΔΔG < threshold decrease affinity. The performance for each method was calculated over the performance on training. mCSM-PPI2 shows a better and more balanced performance on increasing and decreasing affinity mutations than MutaBind.

|  |  | **\|ΔΔG\| > 0 kcal/mol** | | | **\|ΔΔG\| > 0.2 kcal/mol** | | |
| --- | --- | --- | --- | --- | --- | --- | --- |
| **Method** | **Δ Affinity** | **Precision** | **Recall** | **AUC** | **Precision** | **Recall** | **AUC** |
| **mCSM-PPI2** | **Increase** | **0.90** | **0.87** | **0.88** | **0.89** | **0.85** | **0.84** |
|  | **Decrease** | **0.87** | **0.90** | **0.88** | **0.79** | **0.84** | **0.84** |
| **MutaBind** | **Increase** | 0.62 | 0.24 | 0.60* | 0.57 | 0.41 | 0.66* |
|  | **Decrease** | 0.81 | 0.96 | 0.60* | 0.84 | 0.91 | 0.66* |

# * p < 0.05 by z transformation test compared to mCSM-PPI2.

**Table S5** – mCSM-PPI2 training performance comparison across different subsets of variants derived from SKEMPI. The performance of mCSM-PPI2 was calculated after running 10-fold cross-validation 10 times using 90% of the dataset for training and 10% for testing.

| **SKEMPI** | **Method** | **Correlation (ρ)** | **RMSE (kcal/mol)** |
| --- | --- | --- | --- |
| S2007 | **mCSM-PPI2** | **0.83** | **1.02** |
|  | mCSM-PPI1 | 0.80* | 1.25 |
|  | BeAtMuSiC | 0.39* | 1.81 |
| S1964 | **mCSM-PPI2** | **0.82** | **1.08** |
|  | MutaBind | 0.78 | 1.20 |
|  | FoldX | 0.40* | 2.12 |
|  | MMPBSA | 0.44* | 6.45 |
| S1327 | **mCSM-PPI2** | **0.80** | **1.12** |
|  | SAAMBE | 0.62* | NA |
| S1102 | **mCSM-PPI2** | **0.81** | **1.19** |
|  | iSEE | 0.80* | 1.41 |

* p < 0.05 by Fisher r-to-z transformation test compared to mCSM-PPI2.

**Table S6** – Performance of predictive model across the different types of attributes used to build mCSM_PPI2. For this set of experiments the models were trained and performance evaluated on each type of feature separately. The performance was calculated after running 10-fold cross-validation 10 times using 90% of the dataset S8338 and 10% for testing.

| **Type of Feature** | **Correlation (ρ)** | **RMSE (kcal/mol)** |
| --- | --- | --- |
| Graph-based signatures + pharmacophores changes | 0.57 | 2.75 |
| Wild-type residue Environment | 0.28 | 3.13 |
| Nature of Wild-type and Mutant Residues | 0.13 | 4.08 |
| Evolutionary Information | 0.46 | 2.84 |
| Non-Covalent interaction network metrics | 0.38 | 3.62 |
| Energetic Terms | 0.40 | 3.53 |
| Atomic Fluctuation | 0.11 | 5.43 |

**Table S7** – Distribution of mutations over protein-protein complexes for the dataset of alanine-scanning experimental mutations.

| **PDB code** | **Number of mutations** |
| --- | --- |
| 1A22 | 64 |
| 1GC1 | 49 |
| 1DAN | 43 |
| 1JRH | 31 |
| 1BXI | 30 |
| 1A4Y | 28 |
| 1VFB | 28 |
| 3HFM | 25 |
| 1DFJ | 14 |
| 1BRS | 14 |
| 1JCK | 9 |
| 1F47 | 9 |
| 1AHW | 8 |
| 1CBW | 8 |
| 1FCC | 8 |
| 1DN2 | 5 |
| 1FC2 | 3 |
| 2PTC | 1 |
| 1NMB | 1 |

**FIGURES**


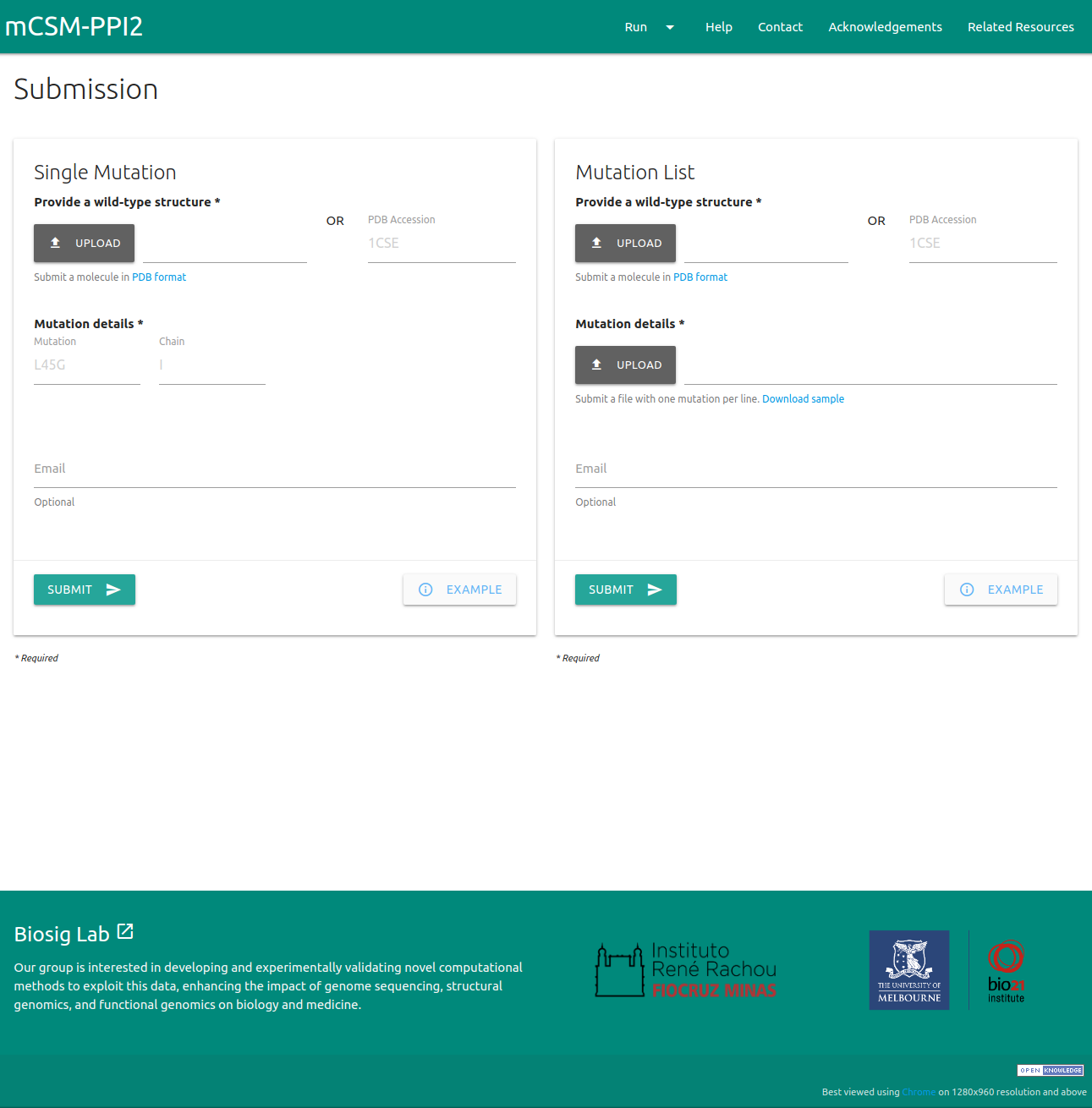


**Figure S1** - Submission page for user-specified mutations. Two options are available. For the “Single Mutation” option mCSM-PPI2 requires one to specify a string containing the wild-type, wild-type residue one-letter code, the position in the structure, the mutant residue one-letter code and the chain identifier. For the “Mutation List” option users are asked to provide a file with a list of mutations for batch processing. For both options a PDB file is also required.


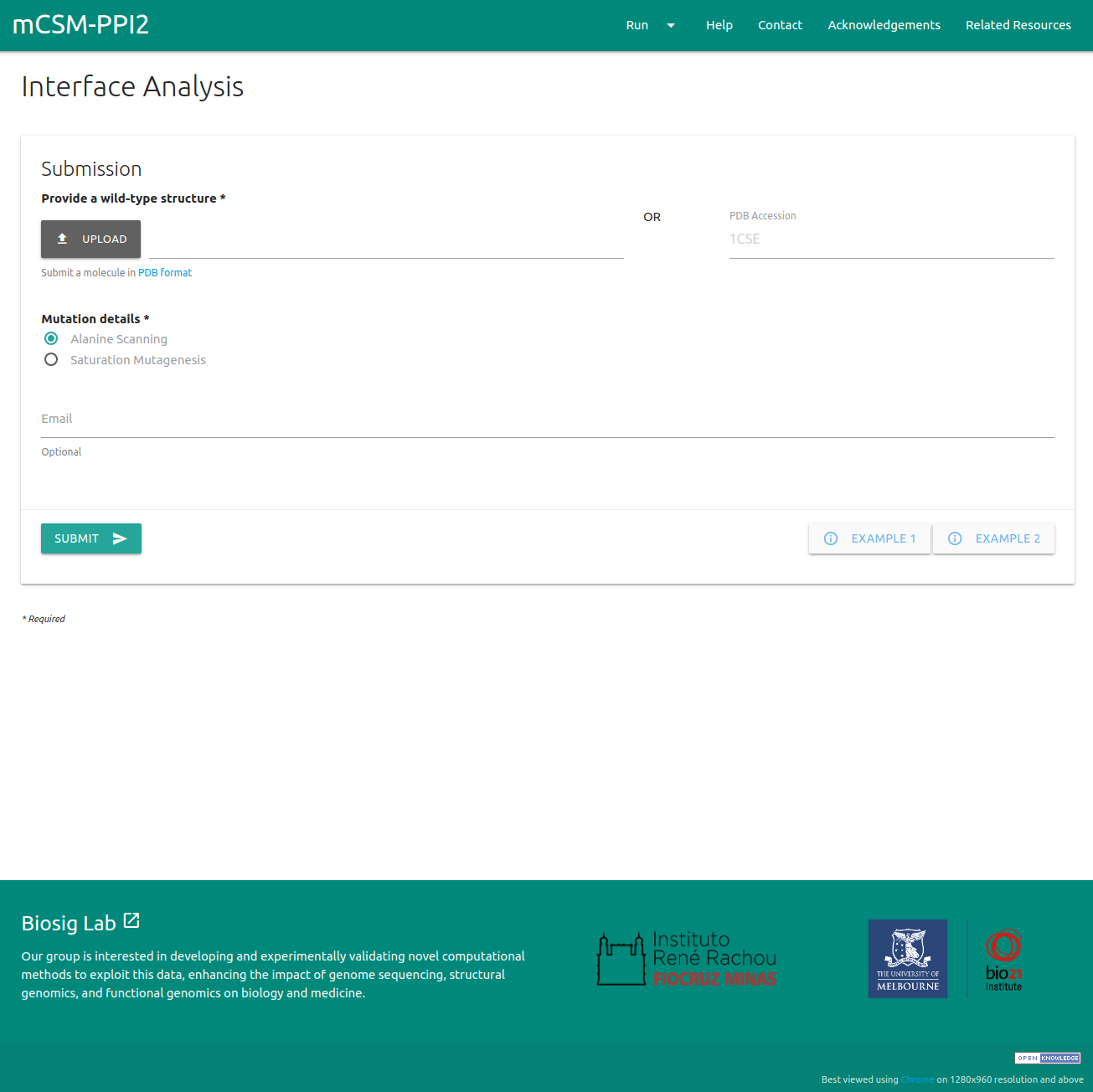


**Figure S2** - Input page for Interface analysis. For assessing the effects of mutations at protein-protein interfaces the server requires the user to provide a PDB file or a PDB accession code and select one of two options: alanine scanning (all interface residues are mutated to alanine) or saturation mutagenesis (all interface residues are mutated to every other amino acid).


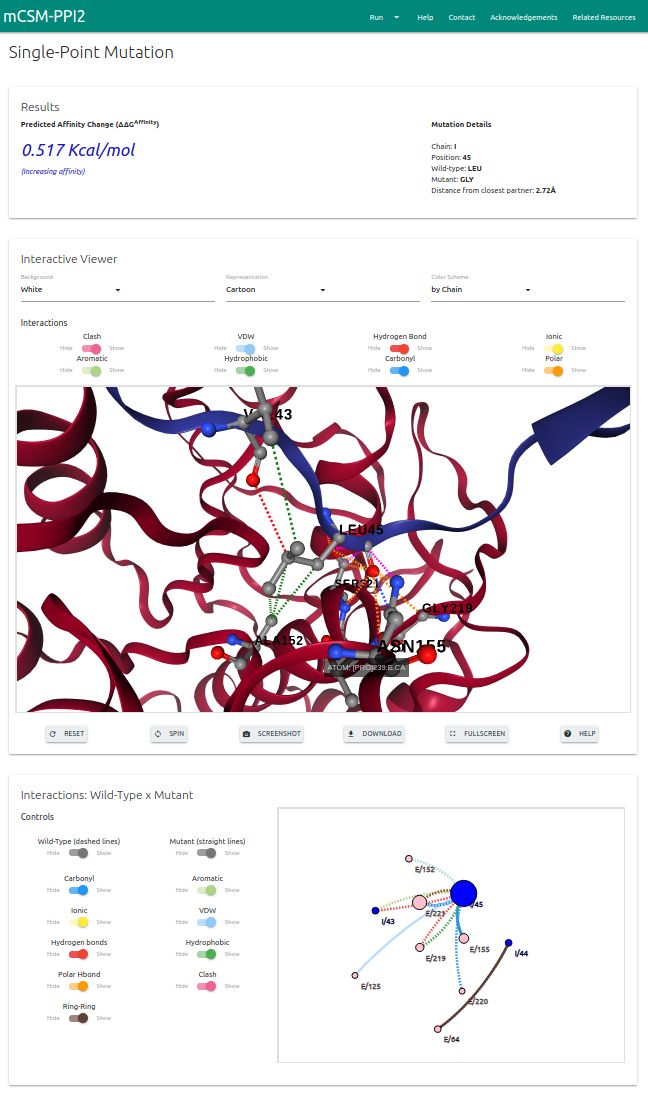


**Figure S3** - Results page for “Single Mutation” option. mCSM-PPI2 outputs the change in binding free energy (in kcal/mol) on the top panel alongside with details on the input mutation. An interactive 3D viewer allows for analysis of non-covalent interactions at the position specified on input. Lastly, a 2D graph displays the interactions of wild-type and mutant residues. In both cases controllers are provided in order to hide or show specific interactions.


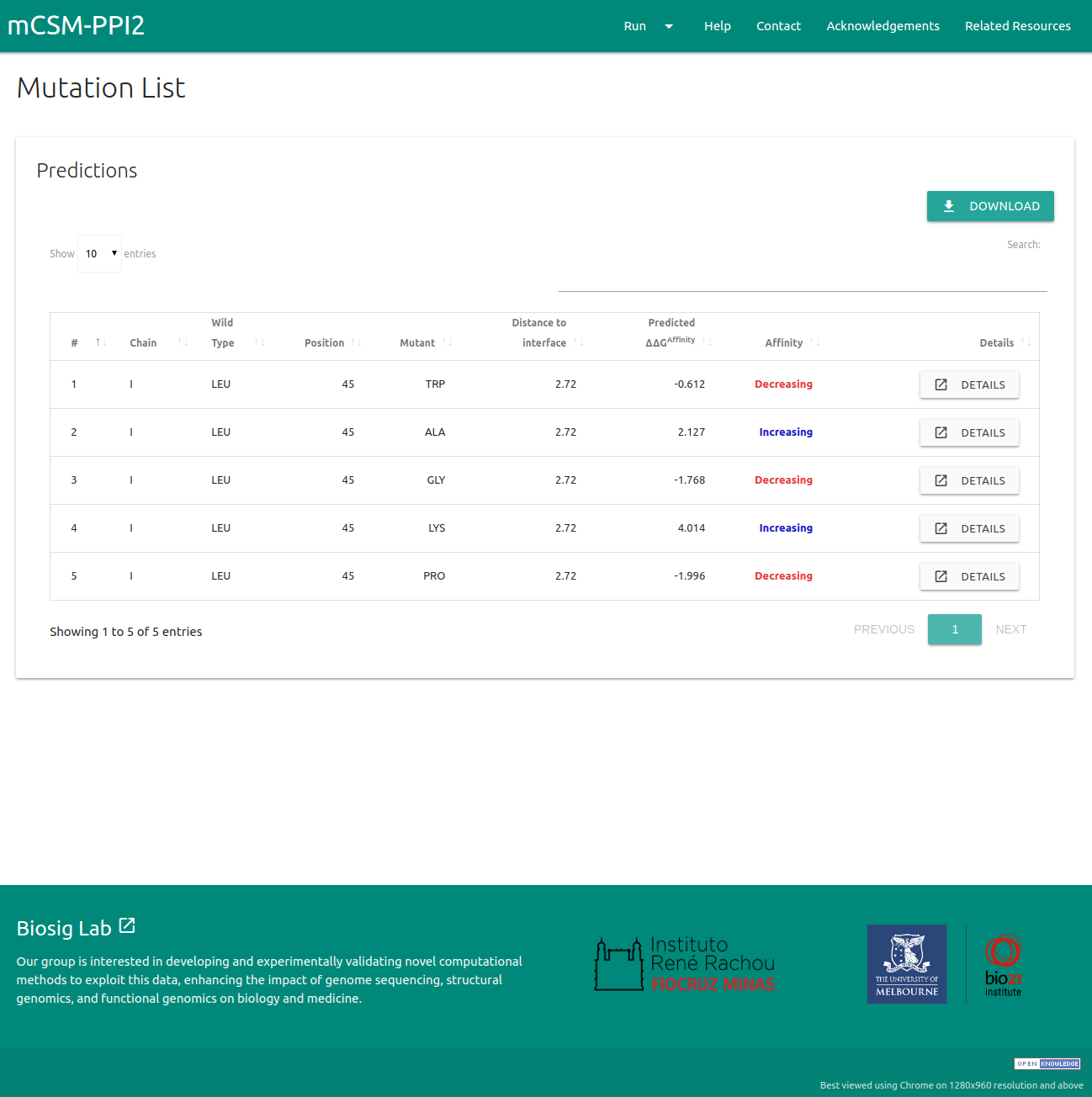


**Figure S4** - Results page for “Mutation List” option. The results are summarised in a downloadable table from which users can access details for each single mutation.


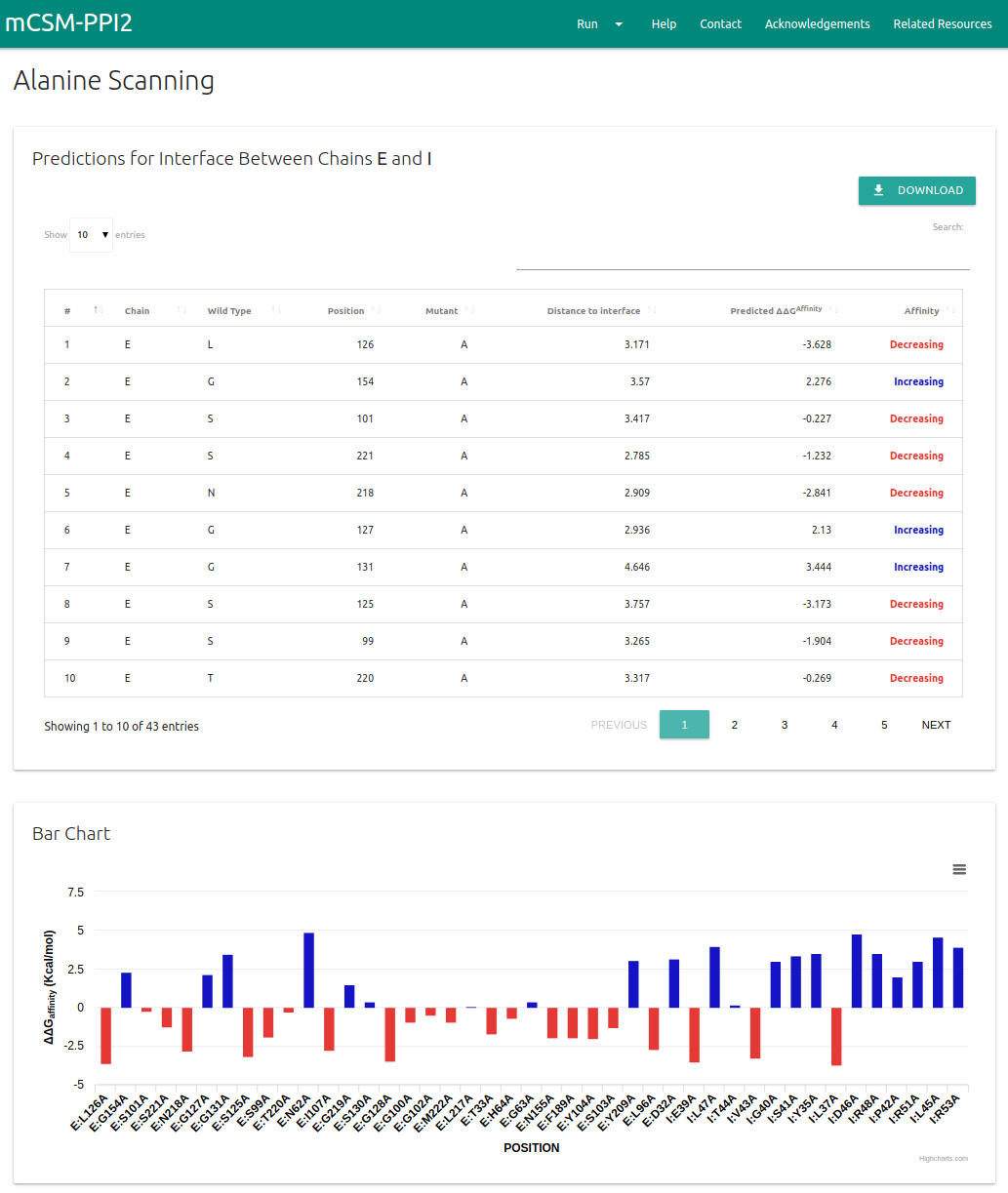


**Figure S5** - Table and Bar graph for Alanine Scanning results page. For each interface identified on the protein complex submitted, mCSM-PPI2 shows a downloadable table with all mutations and the results are also summarised in a bar chart in which the bars are coloured according to the predicted change in binding affinity.


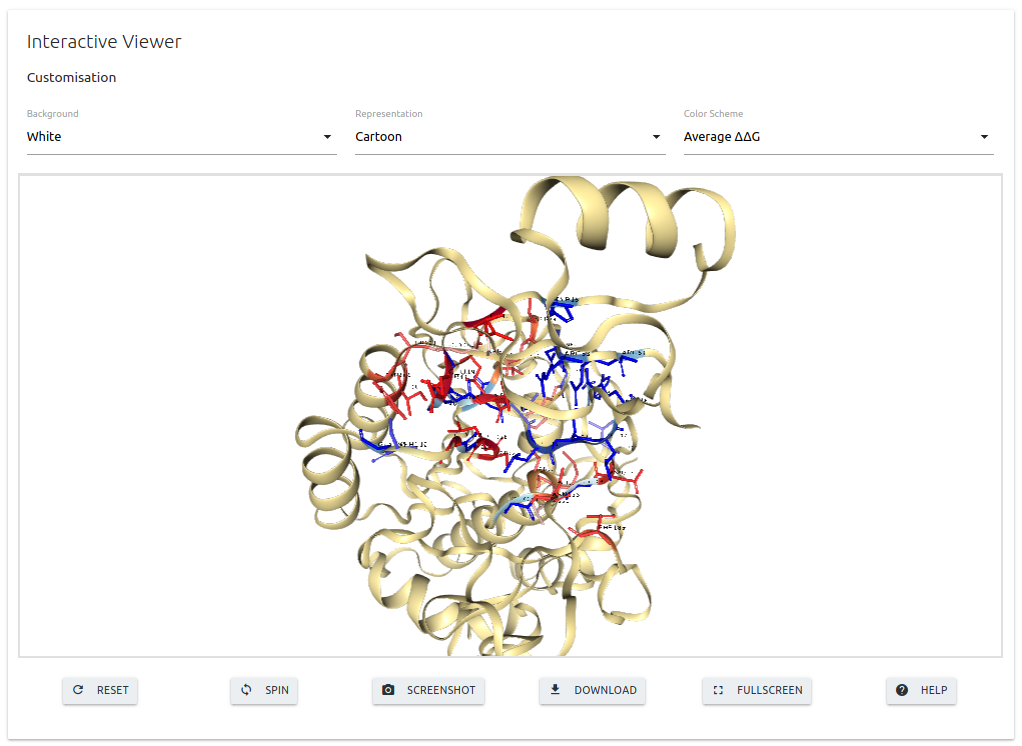


**Figure S6** - 3D viewer for Alanine Scanning results page. A 3D viewer in which interface residues are coloured according to the predicted change in binding affinity is also shown at the bottom of the results page. A set of controllers are available for customising the structure according to the user’s need.


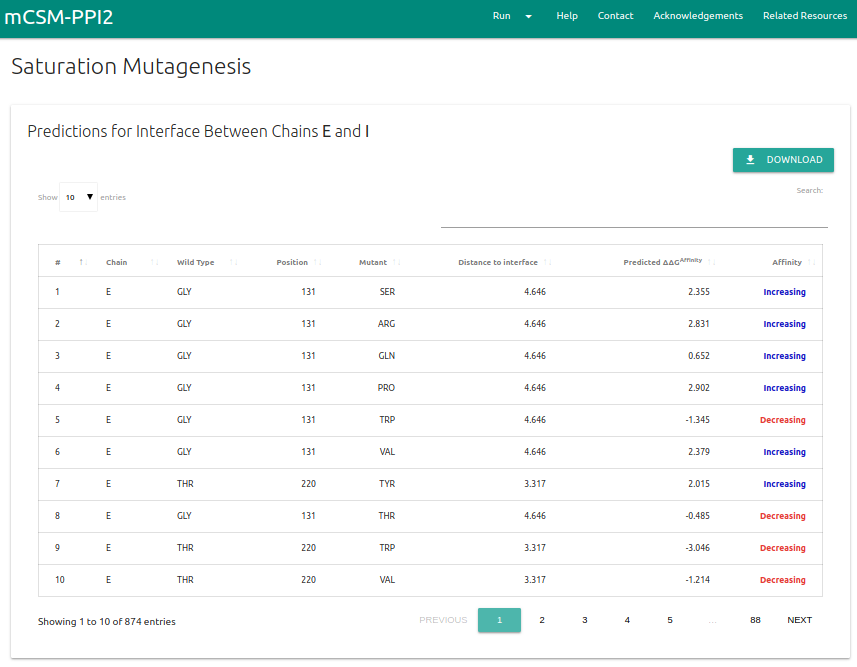


**Figure S7** - Table of results for Saturation Mutagenesis option. Similarly to the “Mutation List” and “Alanine Scanning”, for each interface identified on the Saturation mutagenesis option, the results are compiled in a downloadable table.


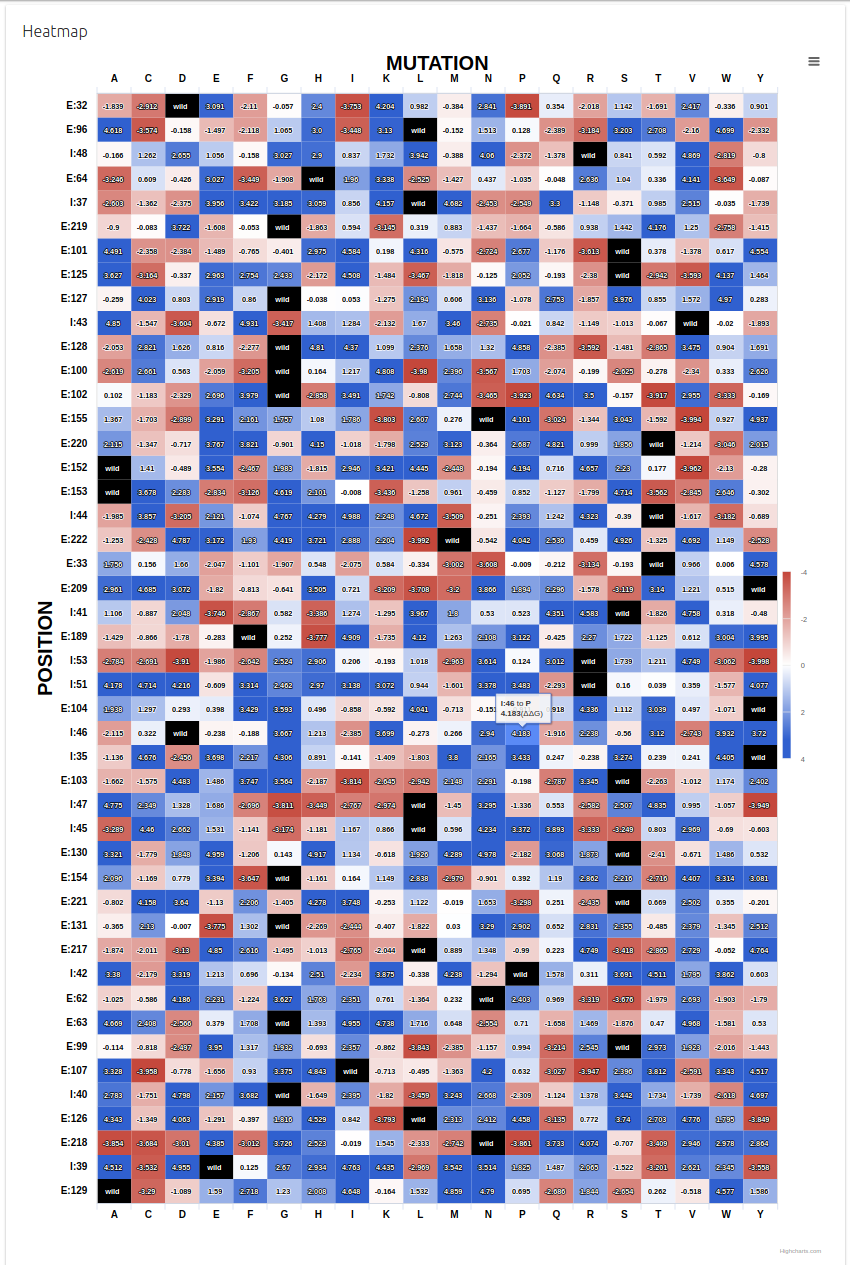


**Figure S8** - Heatmap for Saturation Mutagenesis option. The results compiled in the table for saturation mutagenesis are also summarised in a heatmap in which every mutation is coloured according to the predicted effect.


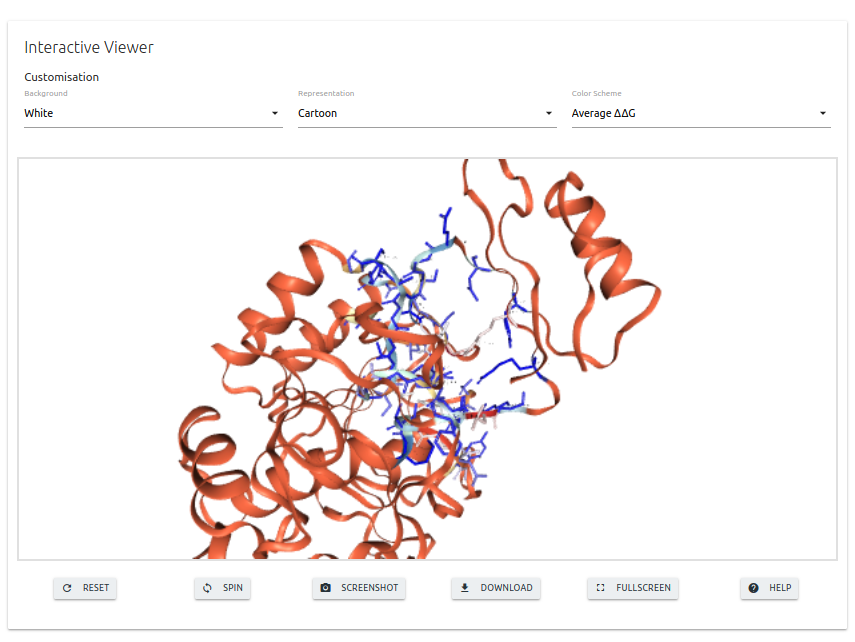


**Figure S9** - 3D viewer for Saturation Mutagenesis results page. mCSM-PPI2 also shows an interactive 3D viewer for the saturation mutagenesis option in which the interface residues are coloured according to the average predicted change in binding affinity.


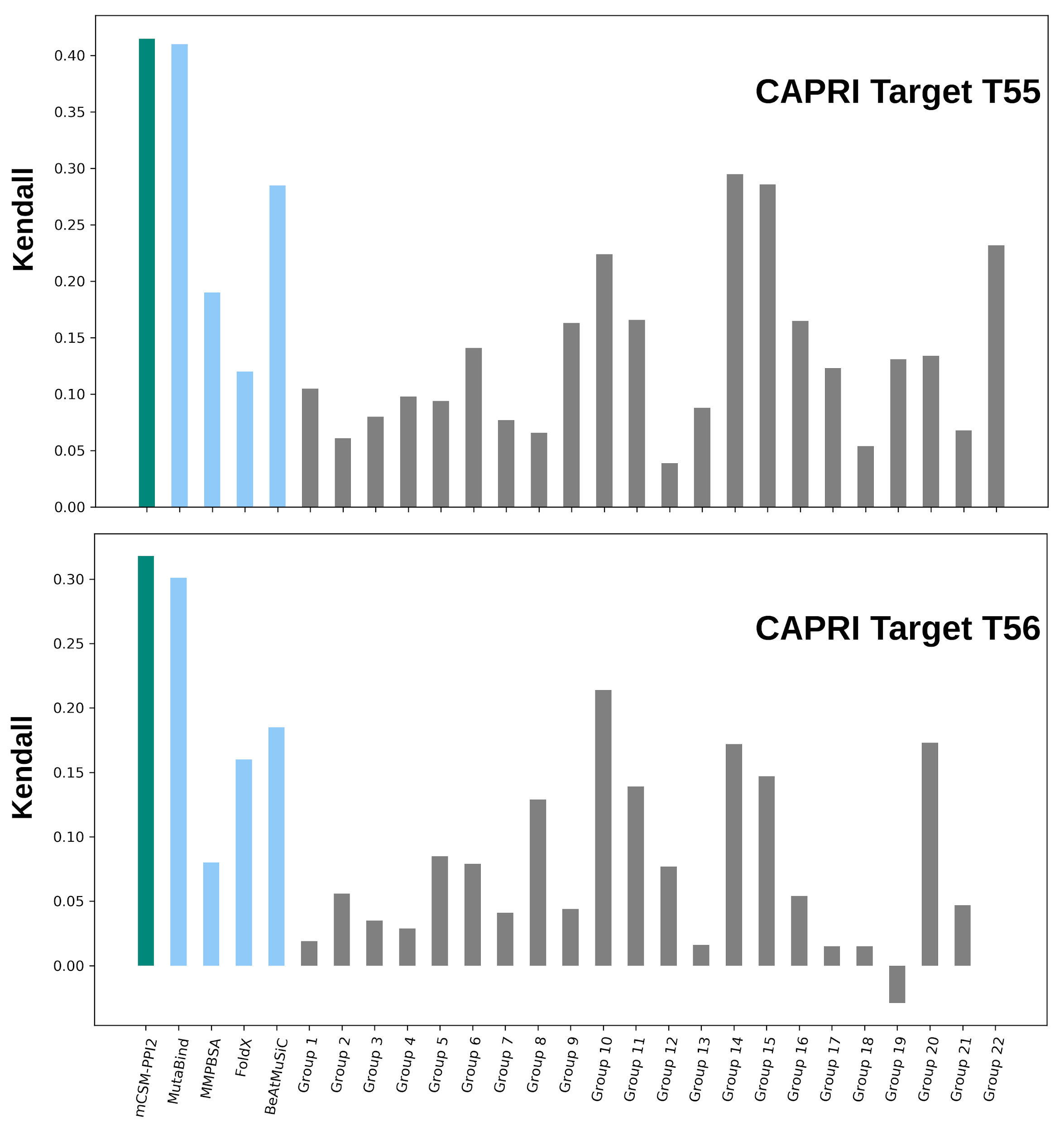


**Figure S10** - Performance comparison on CAPRI round 26th. mCSM-PPI2 (green) outperforms all other 26 methods with a Kendall’s coefficient of 0.42 and 0.32 for datasets based on Targets T55 and T56, respectively. Bar coloured as blue indicate methods that used this same dataset as a benchmark on their studies. Other methods which participated on CAPRI by the time the dataset was released in 2012 were coloured in grey.


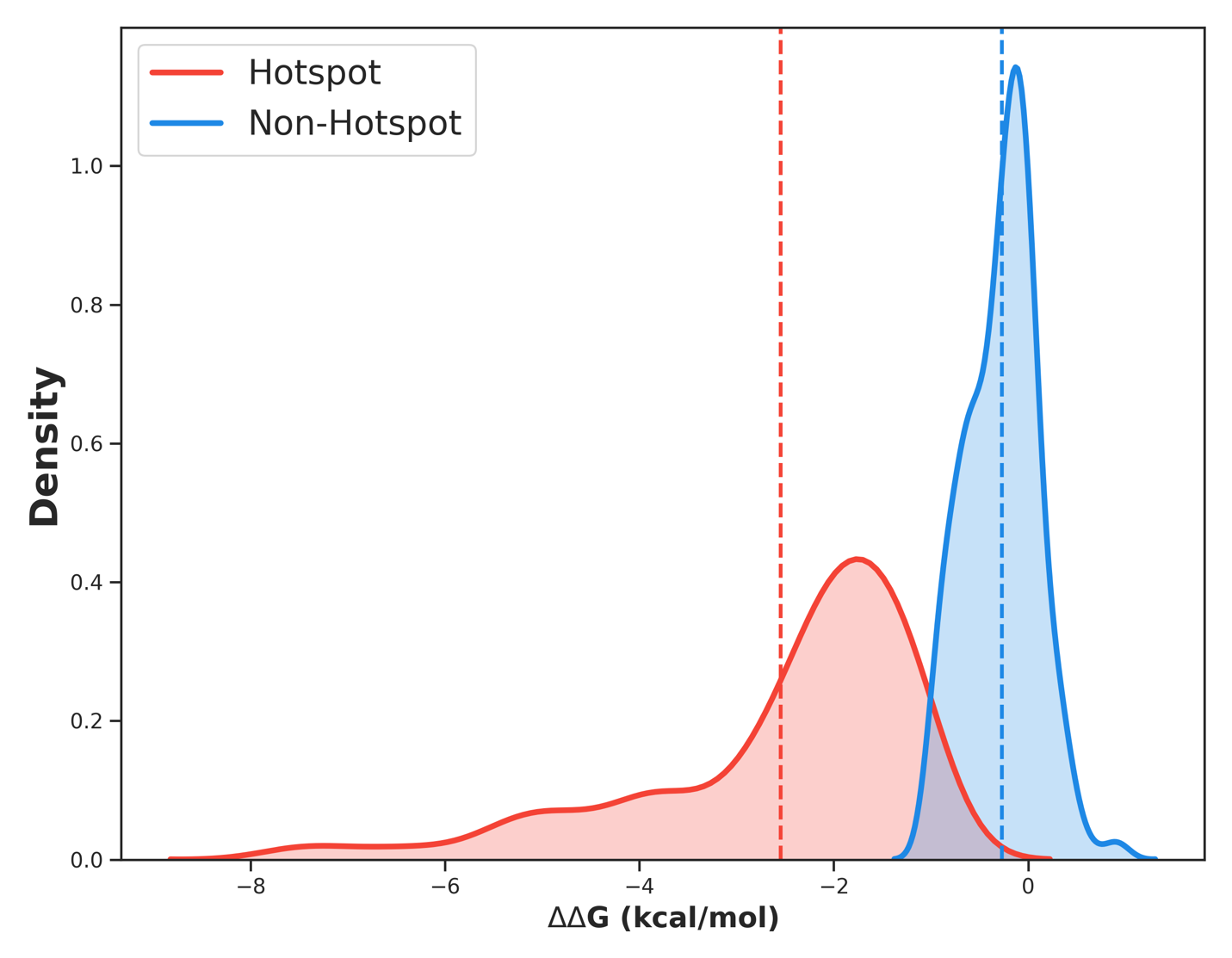


**Figure S11** - Density distribution of ΔΔG predictions by mCSM-PPI2 for mutations that were experimentally assigned as hotspots. While the majority of neutral mutations (blue) were predicted to have little impact, most of hotspots mutations (red) were predicted to have a significant decrease in the binding affinity of the complex.

# REFERENCES

1. Pedregosa, F., Varoquaux, G., Gramfort, A., Michel, V., Thirion, B., Grisel, O., Blondel, M., Prettenhofer, P., Weiss, R., Dubourg, V. *et al.* (2012) *Scikit-learn: Machine Learning in Python*.

2. Geurts, P., Ernst, D. and Wehenkel, L. (2006) Extremely randomized trees. *Machine Learning*, **63**, 3-42.

<http://dx.doi.org/10.1007/s10994-006-6226-1>

3. Sali, A. and Blundell, T.L. (1993) Comparative protein modelling by satisfaction of spatial restraints. *J Mol Biol*, **234**, 779-815.

<http://www.ncbi.nlm.nih.gov/pubmed/8254673>

<http://dx.doi.org/10.1006/jmbi.1993.1626>

4. Fleishman, S.J., Whitehead, T.A., Ekiert, D.C., Dreyfus, C., Corn, J.E., Strauch, E.M., Wilson, I.A. and Baker, D. (2011) Computational design of proteins targeting the conserved stem region of influenza hemagglutinin. *Science*, **332**, 816-821.

<http://www.ncbi.nlm.nih.gov/pubmed/21566186>

<http://dx.doi.org/10.1126/science.1202617>

5. Whitehead, T.A., Chevalier, A., Song, Y., Dreyfus, C., Fleishman, S.J., De Mattos, C., Myers, C.A., Kamisetty, H., Blair, P., Wilson, I.A. *et al.* (2012) Optimization of affinity, specificity and function of designed influenza inhibitors using deep sequencing. *Nat Biotechnol*, **30**, 543-548.

<http://www.ncbi.nlm.nih.gov/pubmed/22634563>

<http://dx.doi.org/10.1038/nbt.2214>

6. Kawashima, S. and Kanehisa, M. (2000) AAindex: amino acid index database. *Nucleic Acids Res*, **28**, 374.

<http://www.ncbi.nlm.nih.gov/pubmed/10592278>

7. Miyazawa, S. and Jernigan, R.L. (1996) Residue-residue potentials with a favorable contact pair term and an unfavorable high packing density term, for simulation and threading. *J Mol Biol*, **256**, 623-644.

<http://www.ncbi.nlm.nih.gov/pubmed/8604144>

<http://dx.doi.org/10.1006/jmbi.1996.0114>

8. Koehl, P. and Levitt, M. (2002) Improved recognition of native-like protein structures using a family of designed sequences. *Proc Natl Acad Sci U S A*, **99**, 691-696.

<http://www.ncbi.nlm.nih.gov/pubmed/11782533>

<http://dx.doi.org/10.1073/pnas.022408799>

9. Zhang, C. and Kim, S.H. (2000) Environment-dependent residue contact energies for proteins. *Proc Natl Acad Sci U S A*, **97**, 2550-2555.

<http://www.ncbi.nlm.nih.gov/pubmed/10706611>

<http://dx.doi.org/10.1073/pnas.040573597>

10. Pires, D.E., Ascher, D.B. and Blundell, T.L. (2014) mCSM: predicting the effects of mutations in proteins using graph-based signatures. *Bioinformatics*, **30**, 335-342.

<http://www.ncbi.nlm.nih.gov/pubmed/24281696>

<http://dx.doi.org/10.1093/bioinformatics/btt691>

11. Cock, P.J., Antao, T., Chang, J.T., Chapman, B.A., Cox, C.J., Dalke, A., Friedberg, I., Hamelryck, T., Kauff, F., Wilczynski, B. *et al.* (2009) Biopython: freely available Python tools for computational molecular biology and bioinformatics. *Bioinformatics*, **25**, 1422-1423.

<http://www.ncbi.nlm.nih.gov/pubmed/19304878>

<http://dx.doi.org/10.1093/bioinformatics/btp163>

12. Chen, Z., Zhao, P., Li, F., Leier, A., Marquez-Lago, T.T., Wang, Y., Webb, G.I., Smith, A.I., Daly, R.J., Chou, K.C. *et al.* (2018) iFeature: a Python package and web server for features extraction and selection from protein and peptide sequences. *Bioinformatics*, **34**, 2499-2502.

<http://www.ncbi.nlm.nih.gov/pubmed/29528364>

<http://dx.doi.org/10.1093/bioinformatics/bty140>

13. Jubb, H.C., Higueruelo, A.P., Ochoa-Montano, B., Pitt, W.R., Ascher, D.B. and Blundell, T.L. (2017) Arpeggio: A Web Server for Calculating and Visualising Interatomic Interactions in Protein Structures. *J Mol Biol*, **429**, 365-371.

<http://www.ncbi.nlm.nih.gov/pubmed/27964945>

<http://dx.doi.org/10.1016/j.jmb.2016.12.004>

14. Schymkowitz, J., Borg, J., Stricher, F., Nys, R., Rousseau, F. and Serrano, L. (2005) The FoldX web server: an online force field. *Nucleic Acids Res*, **33**, W382-388.

<http://www.ncbi.nlm.nih.gov/pubmed/15980494>

<http://dx.doi.org/10.1093/nar/gki387>

15. Grant, B.J., Rodrigues, A.P., ElSawy, K.M., McCammon, J.A. and Caves, L.S. (2006) Bio3d: an R package for the comparative analysis of protein structures. *Bioinformatics*, **22**, 2695-2696.

<http://www.ncbi.nlm.nih.gov/pubmed/16940322>

<http://dx.doi.org/10.1093/bioinformatics/btl461>

16. Jankauskaite, J., Jimenez-Garcia, B., Dapkunas, J., Fernandez-Recio, J. and Moal, I.H. (2019) SKEMPI 2.0: an updated benchmark of changes in protein-protein binding energy, kinetics and thermodynamics upon mutation. *Bioinformatics*, **35**, 462-469.

<http://www.ncbi.nlm.nih.gov/pubmed/30020414>

<http://dx.doi.org/10.1093/bioinformatics/bty635>

17. Moal, I.H. and Fernandez-Recio, J. (2012) SKEMPI: a Structural Kinetic and Energetic database of Mutant Protein Interactions and its use in empirical models. *Bioinformatics*, **28**, 2600-2607.

<http://www.ncbi.nlm.nih.gov/pubmed/22859501>

<http://dx.doi.org/10.1093/bioinformatics/bts489>

18. Dehouck, Y., Kwasigroch, J.M., Rooman, M. and Gilis, D. (2013) BeAtMuSiC: Prediction of changes in protein-protein binding affinity on mutations. *Nucleic Acids Res*, **41**, W333-339.

<http://www.ncbi.nlm.nih.gov/pubmed/23723246>

<http://dx.doi.org/10.1093/nar/gkt450>

19. Li, M., Simonetti, F.L., Goncearenco, A. and Panchenko, A.R. (2016) MutaBind estimates and interprets the effects of sequence variants on protein-protein interactions. *Nucleic Acids Res*, **44**, W494-501.

<http://www.ncbi.nlm.nih.gov/pubmed/27150810>

<http://dx.doi.org/10.1093/nar/gkw374>

20. Petukh, M., Dai, L. and Alexov, E. (2016) SAAMBE: Webserver to Predict the Charge of Binding Free Energy Caused by Amino Acids Mutations. *Int J Mol Sci*, **17**, 547.

<http://www.ncbi.nlm.nih.gov/pubmed/27077847>

<http://dx.doi.org/10.3390/ijms17040547>

21. Geng, C., Vangone, A., Folkers, G.E., Xue, L.C. and Bonvin, A. (2019) iSEE: Interface structure, evolution, and energy-based machine learning predictor of binding affinity changes upon mutations. *Proteins*, **87**, 110-119.

<http://www.ncbi.nlm.nih.gov/pubmed/30417935>

<http://dx.doi.org/10.1002/prot.25630>

22. Kortemme, T. and Baker, D. (2002) A simple physical model for binding energy hot spots in protein-protein complexes. *Proc Natl Acad Sci U S A*, **99**, 14116-14121.

<http://www.ncbi.nlm.nih.gov/pubmed/12381794>

<http://dx.doi.org/10.1073/pnas.202485799>
